# Supplementary material for: The Construction and Exploration of a Comprehensive MicroRNA Centered Regulatory Network in Foxtail Millet (Setaria italica L.)
Source: Front Plant Sci. 2022 May 6;13:848474. doi: 10.3389/fpls.2022.848474 (PMC9121102; doi:10.3389/fpls.2022.848474)
Supplement: Supplementary file 2 [file Data_Sheet_2.zip › Sit-miR529b_Seita.9G087600.1_3424_TPlot.pdf]

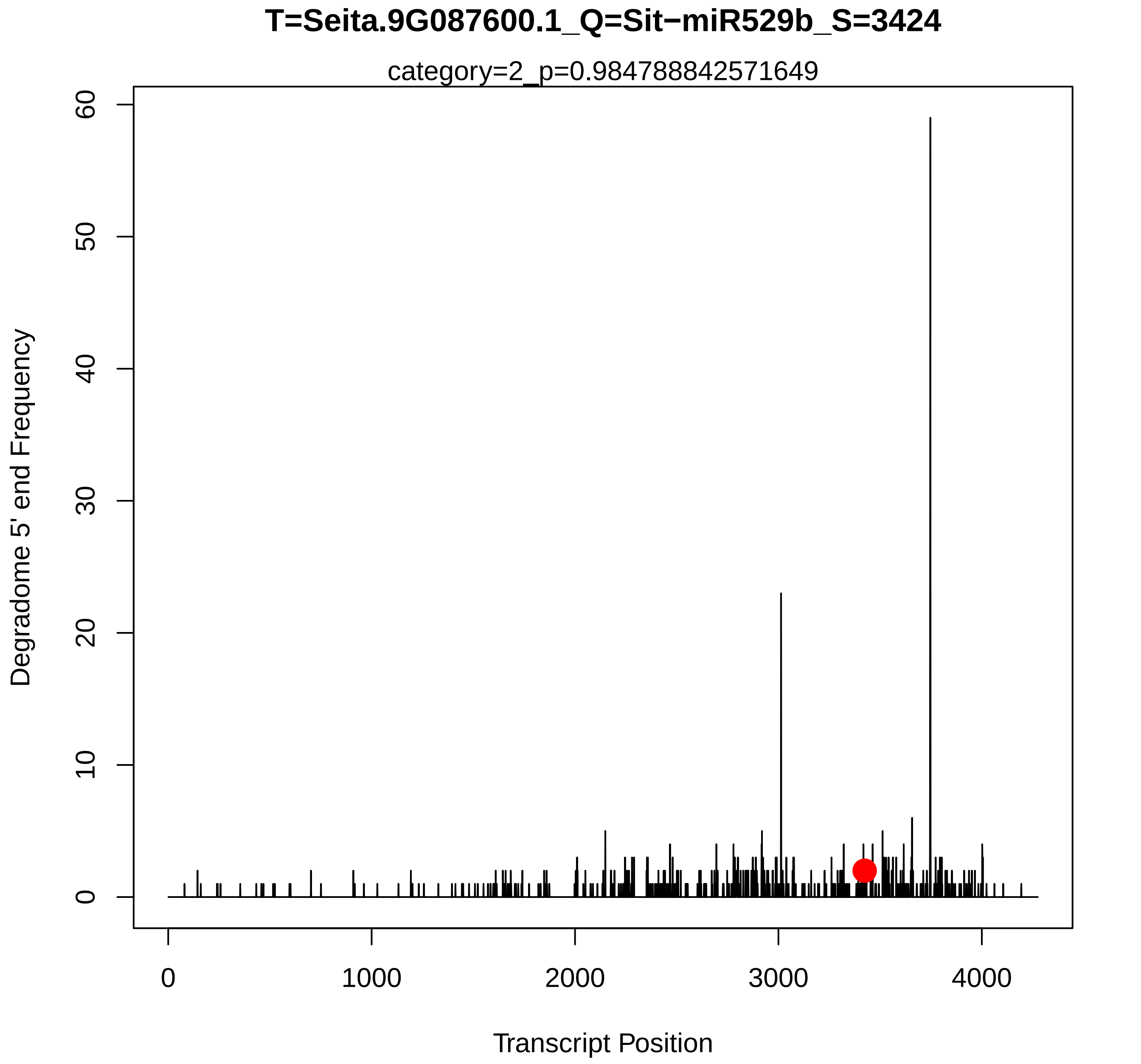
T=Seita.9G087600.1_Q=Sit−miR529b_S=3424
category=2_p=0.984788842571649
Degradome 5' end Frequency
Transcript Position
0
10
20
30
40
50
60
0
1000
2000
3000
4000
